# Impact of septic arthritis on quality of life: arthroscopy vs. arthrotomy

**DOI:** 10.1007/s00402-024-05655-1

**Published:** 2025-03-01

**Authors:** Nike Walter, Lorenz Huber, Melanie Schindler, Josina Straub, Dominik Szymski, Volker Alt, Markus Rupp

**Affiliations:** 1 Department for Trauma Surgery, University Hospital Regensburg, 93053 Regensburg, Germany; 2 Division of Orthopaedics and Traumatology, University Hospital Krems, 3500 Mitterweg 10, Austria; 3 Karl Landsteiner University of Health Sciences, Dr. Karl-Dorrek-Straße 30, Krems, 3500 Austria; 4 Klinik und Poliklinik für Unfall-, Hand- und Wiederherstellungschirurgie Universitätsklinikum Gießen, Rudolf-Buchheim-Straße 7, 35385 Gießen, Germany

**Keywords:** Septic arthritis, Arthroscopy, Arthrotomy, Quality of life, Patient-reported outcome measures

## Abstract

**Introduction:**

Septic arthritis poses significant challenges due to its potential for joint damage and life-threatening complications. The choice between arthroscopy and open arthrotomy as surgical approaches remains a critical decision in septic arthritis management. However, limited research has focused on patient-reported outcomes and quality of life following treatment.

**Materials and Methods:**

A retrospective study was conducted at a German level 1 trauma center, including 58 adult septic arthritis patients treated with arthroscopy (n = 29) or open arthrotomy (n = 29). Quality of life was assessed using the EQ-5D instrument. Functional mobility was evaluated with the Parker Mobility Score, while the Katz Score assessed activities of daily living (ADL). The mean follow-up time was 5.6 years.

**Results:**

Comparable EQ-5D VAS scores were observed in both groups, with no significant difference in the quality of life between arthroscopy and open arthrotomy patients (64.8 ± 19.3 vs. 64.7 ± 19.6, p = 0.749). Notably, both groups reported limitations in pain/discomfort and mobility, while the open arthrotomy group exhibited more anxiety/depression limitations (p = 0.024). Functional mobility, as assessed by the Parker Mobility Score (6.50 ± 2.62 vs. 6.51 ± 2.60, p = 0.617), and ADL independence, using the Katz Score (5.06 ± 1.72 vs. 5.05 ± 1.71, p = 0.181) remained similar between the two groups.

**Conclusion:**

In septic arthritis management, arthroscopy and open arthrotomy yield similar long-term QoL outcomes, functional mobility, and ADL independence. Despite these findings, it is crucial to interpret the results with caution, given potential limitations associated with retrospective studies, and external factors influencing long-term outcomes. Further prospective research, incorporating larger sample sizes and extended follow-up, is necessary to refine our understanding of septic arthritis management strategies and their impact on patient well-being.

**Level of evidence:**

III.

## Introduction

Septic arthritis (SA) refers to an inflammation of joints, typically caused by the invasion of pathogens into the synovial fluid and tissues of a joint. This condition has long posed a significant challenge to both clinicians and researchers due to its rapidly progressing nature and the potential for irreversible joint damage. The risk of receiving arthroplasty within 15 years after SA diagnoses was estimated to be about six times greater than that of the general population [2]. Further, SA is a potentially life-threatening condition with reported 90 days mortality rates between 7.05% and 8.6% [2, 9]. Historically, septic arthritis was described as the “great masquerader” because its clinical presentation can mimic other inflammatory joint diseases, such as rheumatoid arthritis [11]. However, recent developments in diagnostic techniques have greatly enhanced our ability to accurately identify and differentiate septic arthritis from its non-infectious counterparts [16].

Among the critical decisions in its management is the selection of the surgical approach. The two primary strategies include arthroscopy and open arthrotomy. Arthroscopy offers several distinct advantages, including minimal soft tissue disruption, lower rates of readmission, and shorter length of hospital stay [7, 15]. However, arthroscopy may not be suitable for all cases, particularly those with extensive joint involvement or inadequate visualization due to joint effusion. Open arthrotomy entails a larger incision to gain direct access to the infected joint ensuring comprehensive exposure and exploration of the joint. It enables thorough joint irrigation, debridement, and excision of infected tissue. Additionally, open arthrotomy allows for inlay revision, or the change of osteosynthesis material when necessary, which can be crucial in cases where previous hardware or prostheses are involved. This advantage may make open surgery preferable in cases where infection control requires more extensive intervention. The body of evidence comparing the two techniques is still insufficient to reliably conclude that one is the best treatment modality [3].

While numerous studies have extensively examined functional outcomes, such as reinfection and reoperation rates, there is a notable scarcity of studies addressing patient-reported outcomes (PROMs). To date, the impact on quality of life in the context of septic arthritis management has not been evaluated. Therefore, the purpose of this study was threefold aiming at answering the following research questions: (1) What long-term quality of life scores are reported by patients? (2) Are activities of daily living impaired after successful septic arthritis treatment? (3) Is there a difference in PROMS comparing patients treated with arthroscopy and open arthrotomy?

## Methods

A retrospective study of patients treated for septic arthritis was conducted in a level 1 trauma center in Germany. Prior to the start of the study, a positive ethics committee vote was obtained from the ethics committee of the University Hospital Regensburg (file number 20-1681_1-104). This study was carried out in accordance with the Declaration of Helsinki. Informed consent was obtained from all participants. The inclusion period was defined from January 2011 to December 2021. Eligible patients being 18 years or older were screened by international classification of disease (ICD)−10 diagnosis codes “M00.-, septic arthritis”. Afterwards, patients’ medical charts, surgery protocols, laboratory findings as well as microbiological and histopathological reports were screened for confirmation of the diagnoses. Patients with the indication of periprosthetic joint infection or fracture-related infection were excluded. Patient characteristics (sex, age and BMI at the time of surgery) and details of SA (affected joint, Gächter classification [8]) were assessed retrospectively by reviewing electronic medical records. Comorbidities were assessed by obtaining the Charlson Comorbidity Index (CCI) [4]. The minimum clinical follow-up period was set at 24 months. All patients were contacted by telephone.

For the assessment of quality of life, the EQ-5D instrument was employed, a widely recognized and validated measure. The EQ-5D is a standardized questionnaire that assesses health across five dimensions: mobility, self-care, usual activities, pain/discomfort, and anxiety/depression [6]. Additionally, the EQ-5D includes a visual analog scale (VAS) that measures overall health on a scale from 0 to 100, with higher scores indicating better quality of life [1].

To evaluate functional mobility and assess the impact of septic arthritis on physical functioning, the Parker Mobility Score was utilized. This scoring system specifically focuses on the patient's ability to mobilize and perform daily activities. The Parker Mobility Score assigns a numeric value to various mobility aspects, such as walking, standing, and climbing stairs, with higher scores indicating better mobility [13].

To assess the patient’s ability to perform activities of daily living (ADL), the Katz score was used. The Katz Score is a well-established tool that evaluates a patient's independence in six key ADL categories: bathing, dressing, toileting, transferring, continence, and feeding. Each category is scored dichotomously as dependent or independent [10, 17].

### Statistics

Data were analyzed using SPSS statistics version 29.0 (IBM, USA). Descriptive statistics were calculated for all variables. Continuous variables were expressed as the mean and standard deviation (SD). For comparisons between continuous variables, the Mann–Whitney-U test was used after determining the distribution was not appropriate for parametric testing by Levene’s test. Chi-square test was used for comparison of categorical variables. For all tests, *p* values < 0.05 were considered statistically significant.

## Results

**Fig. 1:**
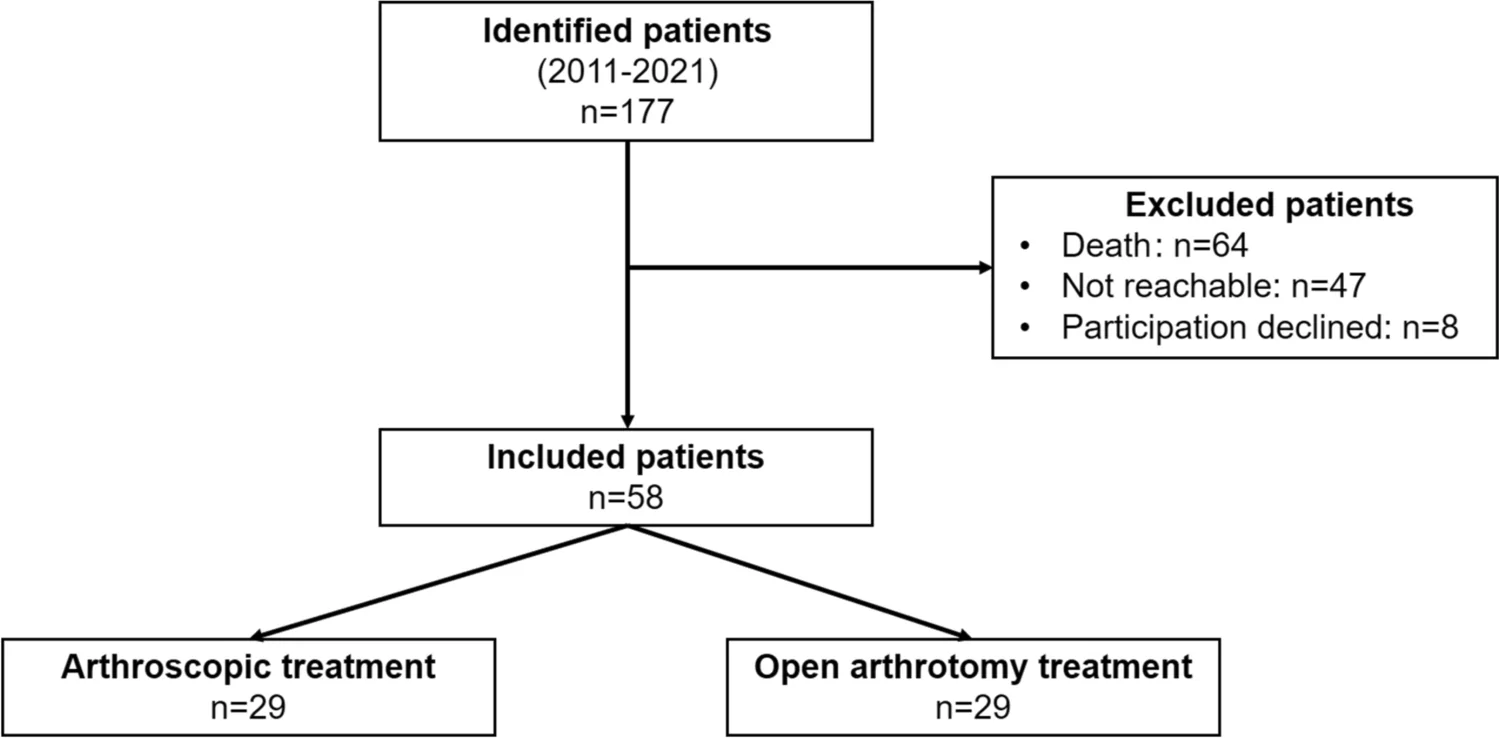
Flowchart of the patient selection. Out of 177 eligible patients treated between 2011 and 2021, n = 58 patients completed the PROM follow-up. Half of the patients were treated with arthroscopic surgery and the other half with open arthrotomy, respectively

Initially, total of n = 177 patients treated for septic arthritis were identified (Fig. 1). Out of these, n = 64 (36.2%) were reported to be dead. Moreover, n = 47 (26.6%) could not be reached as the telephone number was invalid, and n = 8 (4.5%) were not willing to participate in the study. Thus, the final cohort included n = 58 patients (42 male, 16 female). Half of the patients (n = 29) received arthroscopic treatment, whereas in the other half (n = 29 patients) open arthrotomy was performed. The mean follow-up time was 5.6 ± 2.6 years (range 2–9 years). The mean age was 59.8 ± 17.6 years in the arthroscopy group and 63.8 ± 13.1 years in the open arthrotomy group (p = 0.331). In the majority of the patients the affected joint was the knee (48.3 and 41.4%), followed by the shoulder (17.2 and 30.9%) (Table 1).

**Table 1:** Patient demographics

Patients	Arthroscopy n = 29	Open arthrotomy n = 29	p-value
*Sex*			1.000
Male	21 (72.4%)	21 (72.4%)	
Female	8 (27.6%)	8 (27.6%)	
Age	59.8 ± 17.6 years	63.8 ± 13.1 years	0.331
BMI	29.4 ± 7.9 kg/m^2^	29.7 ± 8.1 kg/m^2^	0.593
CCI ASA-Score	1.4 (0–5) 2.5 ± 0.9 (1–4)	1.0 (0–6) 2.5 ± 0.9 (1–4)	0.238 0.978
*Affected joint*			0.066
Knee	14 (48.3%)	12 (41.4%)	
Hip	5 (17.2%)	4 (13.8%)	
Ankle	4 (13.8%)	3 (10.3%)	
Shoulder	5 (17.2%)	8 (30.9%)	
Elbow	1 (3.5%)	1 (3.5%)	
*Gächter classification*			0.78
Stage I	18 (62.1%)	8 (30.9%)	
Stage II	6 (20.6%)	10 (37.8%)	
Stage III	4 (13.8%)	9 (31.0%)	
Stage IV	1 (3.5%)	2 (6.9%)	
Length of hospital stay	26.4 (7–45)	26.0 (9–58)	0.349

*BMI *Body mass index, *CCI* Charlson comorbidity index, *ASA* The American Society of Anesthesiologists Physical Status Classification

**Fig. 2:**
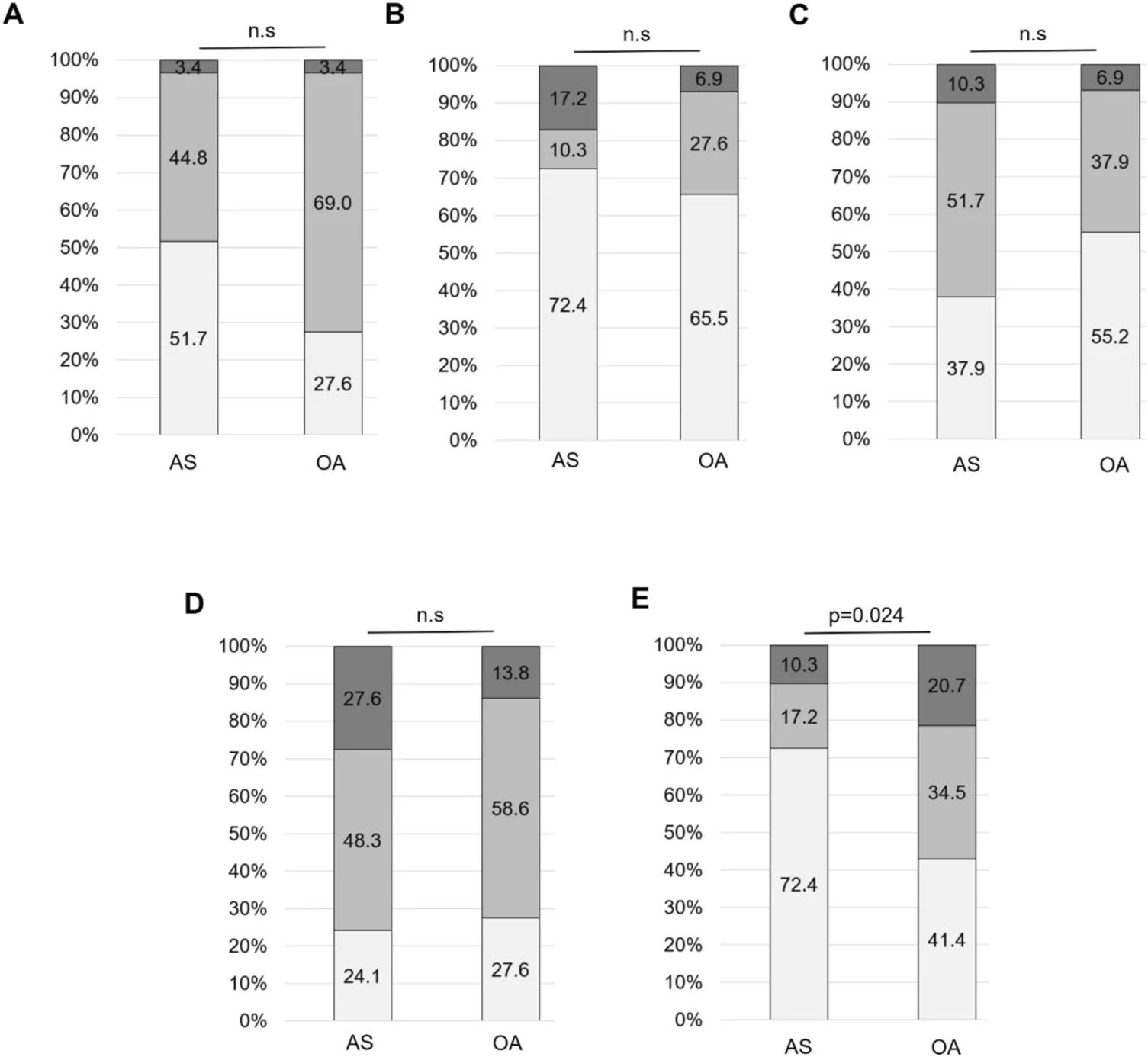
Percentage of patients showing severe, mild, or no limitations in the EQ-5D subdimensions **A** mobility, **B** self-care, **C** usual activities, **D** pain/discomfort, **E** anxiety/depression. *AS* arthroscopic surgery, *OA* open arthrotomy, *n.s.* not significant

The mean EQ-5D VAS rating reached 64.8 ± 19.3 in the arthroscopy cohort compared to 64.7 ± 19.6 in the open arthrotomy group (p = 0.749). In the subdimensions of the EQ-5D, patients in both groups reported limitations, especially concerning pain/discomfort and mobility. The only statistically significant difference was found for anxiety/depression with more limitations in the open arthrotomy group (Fig. 2). The Parker mobility score was comparable (6.50 ± 2.62 vs. 6.51 ± 2.60, 0.617) as well as the Katz ADL total score (5.06 ± 1.72 vs. 5.05 ± 1.71, p = 0.181). There was no statistically significant difference regarding the dimension of the ADL dependence (Table 2).

**Table 2:** Mean values of the parker mobility score, total Katz score and distribution of activities of daily living impairment

Patients	Arthroscopy n = 29	Open surgery n = 29	p- value
EQ-5D VAS	64.8 ± 19.3	64.7 ± 19.6	0.749
Parker mobility score	6.50 ± 2.62	6.51 ± 2.60	0.617
Katz ADL total	5.06 ± 1.72	5.05 ± 1.71	0.181
ADL dependence in			
Bathing	6 (20.7%)	3 (10.3%)	0.302
Dressing	6 (20.7%)	4 (13.8%)	0.525
Toileting	6 (20.7%)	3 (10.3%)	0.302
Transfer	3 (10.3%)	1 (3.4%)	0.317
Continence	6 (20.7%)	6 (20.7%)	0.945
Feeding	3 (10.3%)	0	0.080

*ADL* activities of daily living

## Discussion

The present study sought to address the long-term quality of life, functional limitations in activities of daily living, and differences in PROMs among individuals treated for SA via arthroscopy and open arthrotomy.

Regarding long-term quality of life, as assessed by the EQ-5D instrument, both arthroscopy and open arthrotomy cohorts exhibited comparable EQ-5D VAS scores, with no statistically significant difference. This suggests that, from the patient's perspective, overall health-related quality of life does not significantly differ between the two surgical approaches. Notably, both groups reported limitations in pain/discomfort and mobility. Interestingly, the open arthrotomy group exhibited more limitations in the anxiety/depression dimension. Also, the Parker Mobility Score showed no statistically significant difference between the groups, indicating that both surgical approaches resulted in similar mobility outcomes in the long term. Similarly, the Katz ADL Score revealed no significant differences between the two groups. While there were slight differences in specific ADL impairments (e.g., bathing, dressing, toileting) with a trend towards more ADL impairment in patients treated with arthroscopy, these were not statistically significant. Clinicians can use these findings to provide more informed counseling to septic arthritis patients. They can communicate that, on average, both arthroscopy and open surgery are associated with similar long-term outcomes in terms of functional ability and quality of life, which may help alleviate patient concerns and uncertainties. Furthermore, the surgical therapy decision regarding the patient-centered long-term outcome for or against a therapy form can be facilitated. With equal quality of life in the course, technically surgical and infection treatment-focused influencing factors can be used here for decision-making. The choice between arthroscopy and open arthrotomy in the management of septic arthritis is typically guided by both the severity of the infection and the overall health status of the patient. In our patient cohort, a greater proportion of those undergoing open arthrotomy were classified as Gächter Stages II to IV compared to those treated with arthroscopy (68.9% vs. 37.9%). Although patients treated with arthroscopy had more comorbidities on average, the differences in CCI were not statistically significant. Moreover, patient health status, as assessed by the ASA score, was comparable in both cohorts.

Studies assessing quality of life after SA therapy are scarce. Schmitz and colleagues utilized data from the Swedish Knee Ligament Register demonstrating that patients with SA scored significantly lower on the Knee injury and Osteoarthritis Index Score (KOOS) as well as the EQ-5D two years postoperatively compared to patients, who underwent anterior cruciate ligament reconstruction [14]. In addition, one study conducted in India could be identified assessing PROMs. The authors showed that the EQ-5D scores significantly improved, with the EQ-VAS score raising from 40.6 one months after surgery to a mean of 92.4 after twelve months [12]. This is considerably lower than the scores assessed in the presented study with 64.8 ± 19.3 in the arthroscopy cohort compared to 64.7 ± 19.6 in the open arthrotomy group. The discrepancy might be explainable by differences in age (51.6 ± 13. vs. 59.8 ± 17.6 years and 63.8 ± 13.1 years). However, the age-matched reference from the general German population is 68.6 ± 1.1, which means that our patients reached 94% of the of normative data [5]. Notably, the scores were also better in comparison to periprosthetic joint infection (PJI), where a EQ-VAS value of 52.1 ± 19.6 was reported at a follow up of 4.9 years on average after successful treatment including infection eradication. Additionally, PJI patients reported more limitations in the domain mobility, usual activity, and pain/discomfort [18].

### Limitations

Several limitations must be considered when interpreting our findings. First, the retrospective design of our study introduces potential selection bias and data completeness issues, as data rely on existing medical records. As this is a single-center study conducted in Germany, the generalizability of our findings may be limited by regional differences in demographics and healthcare practices. Furthermore, a substantial proportion of patients were reported as deceased (36.2%), potentially biasing our results toward patients with more severe SA. Patients lost to follow-up or declining participation may also introduce unaccounted-for variations in outcomes or characteristics. The reliance on PROMs introduces subjectivity and recall bias, with the timing of assessments potentially missing evolving changes. In addition, it is important to recognize that the long-term quality of life outcomes observed in this study may be influenced by a myriad of factors beyond the chosen treatment strategy. Over an extended follow-up period of 5.6 years, patient’s lives can undergo significant changes, and various variables, such as new medical conditions, personal life events, and psychosocial factors, may exert substantial effects on QoL. Therefore, while our study provides valuable insights into the impact of arthroscopy and open arthrotomy on QoL, it is prudent to acknowledge that these outcomes are likely multifactorial and not solely attributable to the initial treatment strategy.

## Conclusion

In SA management, arthroscopy and open arthrotomy yield similar long-term QoL outcomes, functional mobility, and ADL independence. Despite these findings, it is crucial to interpret the results with caution, given potential limitations associated with retrospective studies, and external factors influencing long-term outcomes. Further prospective research, incorporating larger sample sizes and extended follow-up, is necessary to refine our understanding of SA management strategies and their impact on patient well-being.

## Data Availability

The data that support the findings of this study are available on request from the corresponding author.
